# Anatomical Laser Microdissection of the Ileum Reveals mtDNA Depletion Recovery in A Mitochondrial Neuro-Gastrointestinal Encephalomyopathy (MNGIE) Patient Receiving Liver Transplant

**DOI:** 10.3390/ijms23158792

**Published:** 2022-08-08

**Authors:** Elisa Boschetti, Leonardo Caporali, Roberto D’Angelo, Carolina Malagelada, Anna Accarino, Maria Teresa Dotti, Roberta Costa, Giovanna Cenacchi, Loris Pironi, Rita Rinaldi, Vincenzo Stanghellini, Stefano Ratti, Lucia Manzoli, Valerio Carelli, Roberto De Giorgio

**Affiliations:** 1 Department of Biomedical and Neuromotor Sciences (DIBINEM), University of Bologna, 40126 Bologna, Italy; 2 IRCCS Azienda Ospedaliero-Universitaria di Bologna, 40126 Bologna, Italy; 3 IRCCS Istituto delle Scienze Neurologiche di Bologna, 40139 Bologna, Italy; 4 Centro de Investigacion Biomedica en Red de Enfermedades Hepáticas y Digestivas (CIBEREHD), University Hospital Vall d’Hebron, 08035 Barcelona, Spain; 5 Departament de Medicina, Universitat Autonoma de Barcelona, 08035 Barcelona, Spain; 6 Department of Medical, Surgical and Neurological Sciences, University of Siena, 53100 Siena, Italy; 7 Department of Medical and Surgical Sciences, University of Bologna, 40126 Bologna, Italy; 8 Department of Translational Medicine, University of Ferrara, 44124 Ferrara, Italy

**Keywords:** gastrointestinal degeneration, microanatomical dissection, mitochondrial disorders, MNGIE, mtDNA depletion

## Abstract

mitochondrial neuro-gastrointestinal encephalomyopathy (MNGIE) is a rare genetic disorder characterized by thymidine phosphorylase (TP) enzyme defect. The absence of TP activity induces the imbalance of mitochondrial nucleotide pool, leading to impaired mitochondrial DNA (mtDNA) replication and depletion. Since mtDNA is required to ensure oxidative phosphorylation, metabolically active tissues may not achieve sufficient energy production. The only effective life-saving approach in MNGIE has been the permanent replacement of TP via allogeneic hematopoietic stem cell or liver transplantation. However, the follow-up of transplanted patients showed that gut tissue changes do not revert and fatal complications, such as massive gastrointestinal bleeding, can occur. The purpose of this study was to clarify whether the reintroduction of TP after transplant can recover mtDNA copy number in a normal range. Using laser capture microdissection and droplet-digital-PCR, we assessed the mtDNA copy number in each layer of full-thickness ileal samples of a naive MNGIE cohort vs. controls and in a patient pre- and post-TP replacement. The treatment led to a significant recovery of gut tissue mtDNA amount, thus showing its efficacy. Our results indicate that a timely TP replacement is needed to maximize therapeutic success before irreversible degenerative tissue changes occur in MNGIE.

## 1. Introduction

Mitochondrial neuro-gastrointestinal encephalomyopathy (MNGIE) is an ultra-rare autosomal recessive disorder due to *TYMP* gene mutations causing a defective thymidine phosphorylase (TP) enzyme [1]. The lack of TP activity induces systemic accumulation of thymidine and deoxyuridine, imbalance of mitochondrial nucleotide pool, and in turn, mitochondrial DNA (mtDNA)-impaired replication and ultimately mtDNA depletion [2]. Since an adequate amount of mtDNA is required for the synthesis of key subunits of oxidative phosphorylation enzymatic complexes, many metabolically active tissues may not reach a sufficient energy production in MNGIE patients [2].

MNGIE is characterized by severe progressive gastrointestinal symptoms and neurological and skeletal muscle impairment [3]. Permanent replacement of TP via allogeneic hematopoietic stem cell (AHSCT) [4,5,6] or liver transplantation (LT) [7,8,9] are the only life-saving approaches to stably reduce nucleoside imbalance, improve skeletal muscle tone, recover walking ability, and re-establish oral feeding [3]. However, the follow-up of patients who have undergone AHSCT or LT showed that gastrointestinal abnormalities and brain leukoencephalopathy never revert [4,7,8,10,11]. Since severe and potentially life-threatening gastrointestinal complications can occur in patients undergoing AHSCT or LT, a thorough assessment of gastrointestinal tract abnormalities pre- and post-TP replacement is crucial for novel therapies.

It was recently demonstrated that the occurrence of microangiopathy is accompanied by massive fibrosis in MNGIE patients’ gut, which persists after stable reintroduction of TP enzymatic activity [12]. However, whether these alterations, which contribute to gastrointestinal worsening, depend on the timing of therapeutic intervention, implying a degree of reversibility of dysfunctional tissue or insufficient mechanistic molecular compensation, remains unsettled. Limited data are available on MNGIE-related mtDNA abnormalities in the different layers of the gastrointestinal tract [13,14,15,16,17], and in particular, whether mtDNA depletion persists or recovers after TP replacement therapies.

Using laser capture microdissection (LCM), this work aimed to characterize the baseline depletion of mtDNA in each layer of the gastrointestinal tract of naive MNGIE patients, assessing also the possible mtDNA copy number changes in the ileum of a patient receiving LT. Our current results provide novel insights on mtDNA recovery after TP replacement therapy, highlighting the clinical/molecular features at follow-up of LT-treated patients, suggesting new important therapeutic implications.

## 2. Results

### 2.1. mtDNA Assessment

**Figure 1:**
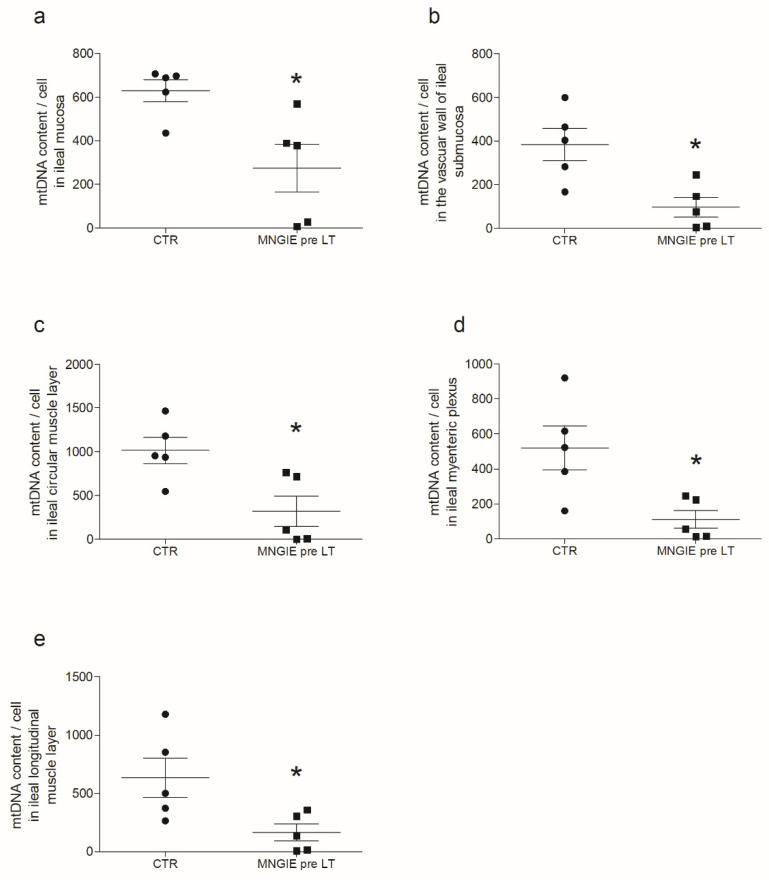
MtDNA depletion in the ileum of MNGIE patients. The figure reports the mtDNA content per cell in the different layers of the ileal wall of control subjects (black circles) vs. MNGIE patient (black squares) specimens. Specifically, the panels are representative of the comparison between controls and MNGIE. Data are reported as scatter plots of mtDNA copy/cell with average ± SD, respectively (**a**) in the mucosa, 630 ± 114 vs. 274 ± 247 (* *p* = 0.0159); (**b**) in the small vessel wall, 383 ± 90 vs. 101 ± 97 (* *p* = 0.0159); (**c**) in circular muscle layer, 1018 ± 338 vs. 320 ± 387 (* *p* = 0.0317); (**d**) in myenteric plexus areas, 521 ± 110 vs. 281 ± 116 (* *p* = 0.0317). No selection of cells present in the ganglion was made; thus, the values refer indistinctly to neuronal, glial, and interstitial cells of Cajal (ICC) which have been obtained from the unavoidable interface between smooth muscle and myenteric plexus; and (**e**) in longitudinal muscle 634 ± 165 vs. 377 ± 162 (* *p* = 0.0317).

Compared to controls, in MNGIE, the mtDNA copy number/cell was reduced by 56% in the mucosa (Figure 1a), 75% in the submucosal small vessel wall (Figure 1b), 69% in the circular muscle layer (Figure 1c), 79% in the myenteric plexus (Figure 1d), and 74% in the longitudinal muscle layer (Figure 1e). No mtDNA deletions were detected in each layer. The mtDNA copy number differed amongst the analyzed areas of the gastrointestinal tract. A higher mtDNA content was located in the internal circular layer. Circular and longitudinal components expressed mtDNA copy number in a 2:1 ratio, and despite the reduction in copy number observed in MNGIE, this difference remained constant. The mucosa in MNGIE showed milder depletion than all other areas analyzed. In control samples, the number of mtDNA copies of the longitudinal muscle layer, mucosa, and myenteric plexus was similar; however, the submucosal vessel wall exhibited a lower mtDNA copy number compared to any other captured areas. The areas displaying the highest mtDNA depletion in MNGIE were the myenteric plexus, vascular wall, and the longitudinal muscle layer.

### 2.2. mtDNA Assessment Pre- vs. Post-LT

**Figure 2:**
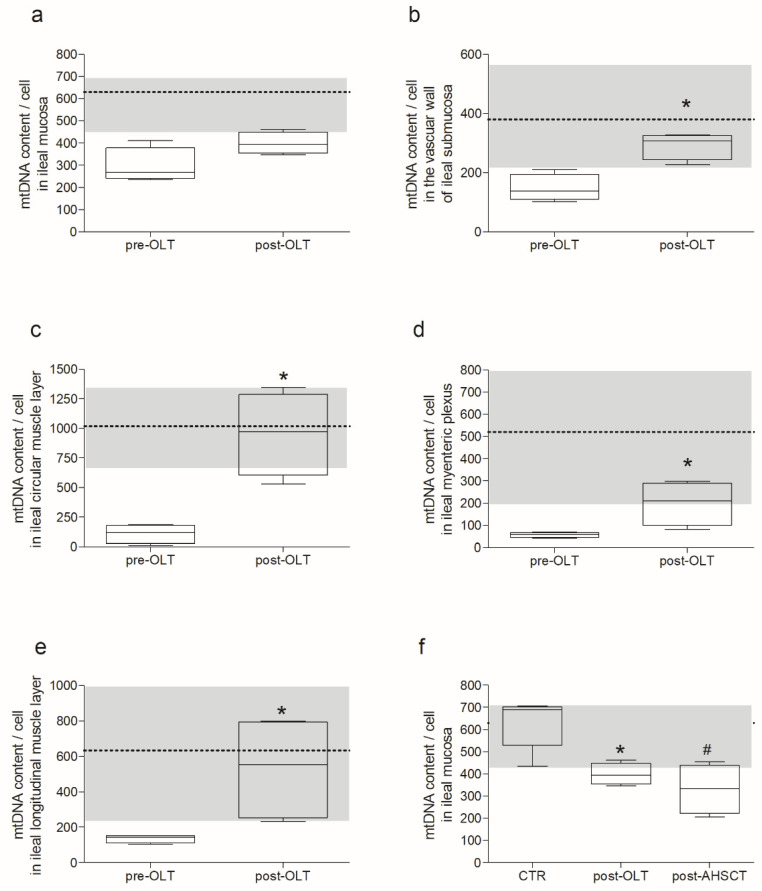
MNGIE patient mtDNA recovery after TP replacement therapy. Each panel (**a**–**e**) reports the mtDNA content per cell in the different layer of the ileal wall of the same patient pre- vs. post-LT. Data are reported as box and whiskers plot, with min to max, of mtDNA copy/cell. Each graph also reports the average of control value, indicated by the dotted line, with ± SD, represented by the grey area. Specifically, the comparison pre- vs. post- LT was performed (**a**) in the mucosa, 296.1 ± 78.3 vs. 399.4 ± 48.8 mtDNA copy/cell ± SD respectively; *p* = 0.2 n.s.; (**b**) in the submucosal small vessel wall, 147.3 ± 45.8 vs. 292.2 ± 45.7 mtDNA copy/cell ± SD respectively; * *p* = 0.0286; (**c**) in the circular muscular layer, 109.1 ± 79.2 vs. 954.0 ± 351.6 mtDNA copy/cell ± SD respectively; * *p* = 0.0286; (**d**) in the myenteric plexus, 56.7 ± 12.3 vs. 200.1 ± 99.6 mtDNA copy/cell ± SD respectively; * *p* = 0.0286; and (**e**) in the longitudinal muscular layer, 136.2 ± 22.4 vs. 533.7 ± 299.3 mtDNA copy/cell ± SD respectively; * *p* = 0.0286. Panel (**f**) is the representation of measurements obtained in the patient post-LT (reported in panel a) in comparison with a patient post-AHSCT (331.7 ± 112.0 mtDNA copy/cell ± SD) vs. control subjects (630 ± 114 mtDNA copy/cell ± SD); *^#^ *p* = 0.0317.

Compared to pre-LT, post-LT samples showed the recovery of the mtDNA depletion in all microdissected areas (Figure 2) except for the mucosa. Despite a trend toward an increased mtDNA copy number (Figure 2a), the difference between pre- and post-LT mucosa was not significant. Furthermore, post-LT values did not reach the range of controls (Figure 2f). The post-LT mtDNA copy captured in submucosal small vessel wall, circular muscle, myenteric plexus, and longitudinal muscle were 2, 8.7, 3.5, and 3.9 times more abundant than pre-LT samples (Figure 2b–e). Apart from post-LT mucosal samples, all other post-LT samples achieved values comparable to those of controls. No mtDNA deletions were detected pre- or post-LT.

### 2.3. mtDNA Assessment Post-AHSCT

The mtDNA copy number/cell was measured in a mucosal sample of the ileum of a MNGIE patient 4 years post-AHSCT (Figure 2F). These data showed no significant difference with those obtained 6 months post-LT (*p* = 0.8467), but mtDNA amount was still depleted compared to controls (*p* = 0.0317).

## 3. Discussion

This study provides the first detailed analysis of the mtDNA copies *per cell* in the different layers of the ileum of a MNGIE patient pre- and post-LT, a therapeutic procedure providing a stable source of TP. A significant recovery of mtDNA amount was observed after LT, providing a proof of concept for the molecular therapeutic efficacy with clear-cut implications on the importance of a timely LT intervention to maximize therapeutic success. Since mtDNA copies/cell vary between cell types and tissues [18], mtDNA content/cell of five controls was determined with a laser microdissection technique coupled to ddPCR, providing a distribution map of mtDNA amounts in the ileum.

The mtDNA depletion in MNGIE is driven by the excess of circulating nucleosides. Specifically, thymidine and cytidine are both substrates of thymidine kinase 2, a key enzyme of the mitochondrial salvage pathway. In MNGIE, the excess of thymidine enters mitochondria in a non-equimolar competition with cytidine for the phosphorylation operated by thymidine kinase 2. This leads to a greater incorporation of thymine as compared to cytosine. The resulting cytosine deficiency induces a progressive mtDNA depletion, which impairs the mitochondrial salvage pathway, leading to progressive mtDNA depletion [19]. The cellular maintenance of mtDNA content is a dynamic and tightly regulated process [20]. However, tissue-specific mtDNA copy number control mechanisms are still unclear [18]. Furthermore, variations in mtDNA levels often accompany key pathophysiological changes characterizing the transition to the disease state [21]. Herein, it was shown that the clearance of circulating nucleosides obtained by LT is sufficient to restore gut mtDNA copy number similar to controls. As previously documented, the circulating nucleosides measured in plasma drop down in 24 h post-LT [8]. The recovery of the mtDNA depletion was demonstrated in both muscle layers, the myenteric plexus, and the wall of submucosal blood vessels six months post-LT. In MNGIE gut mucosa, the same trend toward increased mtDNA copy number was observed, but without reaching the control values. Thus, it is conceivable that more than six months are needed to achieve a full recovery. However, we measured the mtDNA copy number of ileal mucosal biopsies from a patient who had received AHSCT 4-years prior sampling [22], and the result was comparable to the values so far obtained six-month-post-LT. Thus, the reasons why the mucosa, which is the least mtDNA-depleted layer, does not fully recover the mtDNA copy number post-LT need future elucidation.

Our previous studies demonstrated ultrastructural changes in MNGIE gastrointestinal tract that did not revert post-LT [12]. Compared to controls, patients with MNGIE showed a decreased area of vascular tissue but a twofold increase of submucosal vessels/mm^2^ with small size, whereas medium and large size vessels were decreased. Small vessels, due to the absence of TP activity, were mainly generated by vascular endothelial growth factor signalling, thus prone to disruption. Fibrosis index and hypoxia-inducible factor 1-alpha protein expression were both increased, suggesting tissue hypoxia, which increased from mucosa to the longitudinal muscle layer. In addition to a reduced thickness of the longitudinal muscle layer, we also detected an increased inter-ganglionic distance and a reduced number of myenteric neurons. In this study, the post-LT mtDNA copy number reverts to control levels, and this appears to be the only gastrointestinal parameter normalized by LT. Our results strongly suggest that an early TP replacement, bringing the cellular mtDNA content back to normal levels, could prevent or slow down MNGIE-related irreversible fibrotic alterations. Whether the observed effects are primarily due to the absence of TP, to the prolonged exposure to nucleosides resulting in mtDNA depletion, or to a combination of both remains unsettled.

## 4. Materials and Methods

### 4.1. Patients and Sampling

Ileal biopsies (1 × 1 cm) were obtained from *n* = 5 MNGIE patients (1 female; 20–38 years) and *n* = 5 controls (1 female; 32–55 years) without symptoms suggestive of gastrointestinal impairment. Specifically, *n* = 3 controls underwent abdominal surgery and *n* = 2 were obtained from human cadavers without any gastrointestinal-associated pathology from “*donors of the body donation program*” of the University of Bologna. A full-thickness ileal biopsy was collected from the first MNGIE patient receiving LT (pre- and post-LT). Specimens were fixed in 4% formalin (Merck KGaA, Darmstadt, Germany) and embedded in paraffin (Merck KGaA, Darmstadt, Germany). One mucosal biopsy from the ileum was endoscopically collected from a MNGIE patient (1 female; 27 years) 4 years post-AHSCT.

### 4.2. Clinical History and Pre- and Post-LT Tissue Sampling of Patient #1

The clinical history of the first patient who underwent LT, a 25-year-old man carrying the homozygous c.1160-1G > A/*TYMP* mutation, was previously reported [7,8]. Notably, his plasma TP activity was markedly reduced (4 nmol/h* mg; n.v.: >253) along with increased levels of dThd and dUrd (2.87 μg/mL and 3.73 μg/mL, respectively; *n*. values: undetectable). LT was performed when the patient was in critical condition with a clinical picture characterized by relapsing fever, daily vomiting, severe abdominal pain, malnutrition, inability to walk, and bed-restriction with lower-limb hyposthenia. He received a temporary decompressive ileostomy during LT by which a pre-LT biopsy sample (1 × 1 cm) was obtained; eight months later, during the ileostomy closure, a second biopsy (1 × 1 cm) was collected as post-LT sample. Although the patient progressively improved, as demonstrated by the recovery of oral feeding, lower-limb strength, walking, and driving ability, no weight gain was noted and thirty-two months after LT he developed massive gastrointestinal bleeding, causing death.

### 4.3. LCM of Ileal Samples

**Figure 3:**
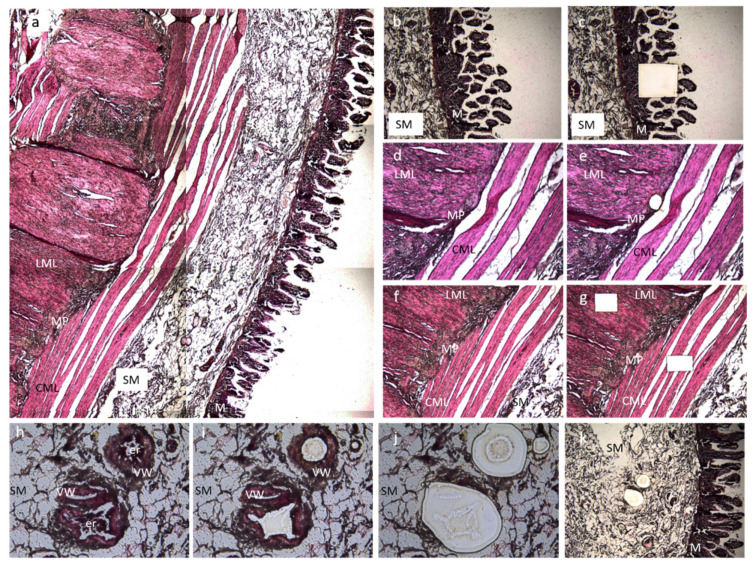
Microdissection of ileal samples. The picture is representative of an ileal section of an MNGIE patient stained with hematoxylin and eosin pre- and post-microdissection. (**a**) Full thickness ileal sample; from the right to the left, we can appreciate the mucosa (M), the submucosal layer (SM), the circular muscular layer (CML), the myenteric plexus (MP), and the longitudinal muscle layer (LML). Image was captured using 5× magnification. (**b**) Pre- and (**c**) post-laser capture of a mucosal sample. Both images were captured using 5× magnification. (**d**) Pre- and (**e**) post-laser capture of the myenteric plexus. Both images were captured using 10× magnification. (**f**) Pre- and (**g**) post-laser capture of longitudinal and circular muscle layers samples. Both images were captured using 5× magnification. (**h**–**k**) Microdissection of the small vessel wall. (**h**) Picture representative of the three selected vessels (20× magnification). (**i**) A first dissection was made to clean the inner part of the vessel from circulating cells. The captured section is discarded (20× magnification). (**j**) Collection of the section containing the vascular wall (20× magnification). (**k**) Post-laser capture of vascular wall in the submucosa (10× magnification). All microdissected sections were separately collected onto the caps of 0.2 mL PCR tubes, and DNA was extracted on the same day of collection.

Formalin fixed-paraffin embedded specimens from each subject and control were sectioned at 10 µm and placed on 2.0 µm nuclease and human nucleic acid free polyethylene naphthalate (PEN) membrane slides (Leica Microsystems; Milano, Italy). After deparaffination and rehydration, sections were stained with hematoxylin and eosin (Merck KGaA, Darmstadt, Germany) for histological recognition of the different gut layers/cell types. Based on morphological criteria, each ileal layer (mucosa; submucosal small vessels deprived of any blood cells; circular muscle layer, myenteric plexus and longitudinal muscle layer) were microdissected (Figure 3) using Leica LMD 6 Laser Capture Microdissection system (Leica Microsystems; Milano, Italy) equipped by a Leica DFC7000 T camera. Microdissected sections were separately collected onto the caps of 0.2 mL PCR tubes (Merck KGaA, Darmstadt, Germany) and DNA were extracted on the same day of collection.

### 4.4. Total DNA Extraction and mtDNA Copy Number and Deletion Quantification

Total DNA (nuclear and mitochondrial) was extracted in a solution containing 50 mM TRIS HCl pH8.5; 6.9 μM proteinase K (Cell Signaling Technology, Danvers, MA, USA) and 0.1%TWEEN 20 (Merck KGaA, Darmstadt, Germany) and assessed via a thermal cycler for 16 h 55 °C and 10 min 99 °C. The absolute quantification of mtDNA copy number and deletion was performed using droplet digital PCR (ddPCR). The mtDNA copy is based on duplex amplification with specific probes of nuclear and mitochondrial DNA and expressed as a ratio of mtDNA and nDNA multiplied by two [23]. The mtDNA primers used were designed on *MT-ND2* gene: mtDir (CACAGAAGCTGCCATCAAGTA), mtRev (CCGGAGAGTATATTGTTGAAGAG) with probe: (FAM/CCTCACGCAAGCAACCGCATCC/BHQ1). The primers for nuclear gene GenDir were designed on *FASLG* gene: GenDir(GGCTCTGTGAGGGATATAAAGACA), GenRev (CAAACCACCCGAGCAACTAATCT) with probe: (HEX/CTGTTCCGTTTCCTGCCGGTGC/BHQ1). The mtDNA deletions quantification was based on duplex amplification with specific probes in MT-ND4 (major arc) and MT-ND1 (control region) and expressed as ratio ND4/ND1 [24]. The *MT-ND4* primers used were: ND4Fdel (CCATTCTCCTCCTATCCCTCAAC), ND4Rdel (CACAATCTGATGTTTTGGTTAAACTATATTT), with probe: (HEX/CCGACATCATTACCGGGTTTTCCTCTTG/BHQ1); the MT-ND1 primers used were: ND1Fdel (CCCTAAAACCCGCCACATCT), ND1Rdel (GAGCGATGGTGAGAGCTAAGGT), with probe: (6FAM/CCATCACCCTCTACATCACCGCCC/BHQ1). The reaction was performed using ddPCR Supermix for Probes (No dUTP) (Bio-Rad, Milano, Italy) following the manufacturer instructions and 1:2 diluted DNA in molecular-grade water. The analysis was carried out on QX200 ddPCR System (Bio-Rad). Data were analyzed with Quantasoft Analysis Pro 1.0 (Bio-Rad, Milano, Italy).

### 4.5. Statistic

The statistical analysis was performed using GraphPad Prism Software 5.0; Mann–Whitney test was used for group comparisons.

## 5. Conclusions

This study provides the first evidence that gastrointestinal mtDNA content may recover after LT, supporting the possibility that the gut may contribute to improve and potentially revert the clinical picture. The persistent alterations observed in contrast with mtDNA recovery are possibly due to the advanced state of disease occurring at the time of LT. This again suggests that LT should be performed as early as possible to maximize the full recovery of the mtDNA content and reversible histopathological changes. Indeed, the impact of MNGIE-related gastrointestinal pathophysiology remains the therapeutic challenge for MNGIE patients.

## Data Availability

The datasets used and/or analyzed during the current study available from the corresponding author on reasonable request.

## Abbreviations

AHSCT	Allogeneic Hematopoietic Stem Cell
ddPCR	droplet digital PCR
LCM	Laser Capture Microdissection
LT	Liver Transplantation
mtDNA	Mitochondrial DNA
MNGIE	Mitochondrial NeuroGastroIntestinal Encephalomyopathy
TP	Thymidine Phosphorylase
